# Simultaneous expression of an endogenous spermidine synthase and a butanol dehydrogenase from *Thermoanaerobacter pseudethanolicus* in *Clostridium thermocellum* results in increased resistance to acetic acid and furans, increased ethanol production and an increase in thermotolerance

**DOI:** 10.1186/s13068-023-02291-6

**Published:** 2023-03-14

**Authors:** Sun-Ki Kim, Yannick J. Bomble, Janet Westpheling

**Affiliations:** 1 Department of Genetics, University of Georgia, Athens, GA 30602 USA; 2 Oak Ridge National Laboratory, The BioEnergy Science Center and The Center for Bioenergy Innovation, Oak Ridge, TN 37831 USA; 3 Department of Food Science and Technology, Chung-Ang University, Anseong, Gyeonggi 17546 Republic of Korea; 4 Biosciences Center, National Renewable Energy Laboratory, Golden, CO USA

**Keywords:** Inhibitor tolerance, Biofuels, *Clostridium thermocellum*, Spermidine, Butanol dehydrogenase

## Abstract

**Background:**

Sensitivity to inhibitors derived from the pretreatment of plant biomass is a barrier to the consolidated bioprocessing of these complex substrates to fuels and chemicals by microbes. Spermidine is a low molecular weight aliphatic nitrogen compound ubiquitous in microorganisms, plants, and animals and is often associated with tolerance to stress. We recently showed that overexpression of the endogenous spermidine synthase enhanced tolerance of the Gram-positive bacterium, *Clostridium thermocellum* to the furan derivatives furfural and HMF.

**Results:**

Here we show that co-expression with an NADPH-dependent heat-stable butanol dehydrogenase from *Thermoanaerobacter pseudethanolicus* further enhanced tolerance to furans and acetic acid and most strikingly resulted in an increase in thermotolerance at 65 °C.

**Conclusions:**

Tolerance to fermentation inhibitors will facilitate the use of plant biomass substrates by thermophiles in general and this organism in particular. The ability to grow *C. thermocellum* at 65 °C has profound implications for metabolic engineering.

**Supplementary Information:**

The online version contains supplementary material available at 10.1186/s13068-023-02291-6.

## Background

Pretreatment of plant biomass releases several inhibitors that adversely affect the growth of many microorganisms including furans and acetic acid. The generation of acetic acid is an inevitable consequence of microbial fermentations and unlike furans, furfural and HMF, acetic acid is generated by the hydrolysis of acetylxylan found in hemicellulose regardless of the pretreatment method. Moreover, concentrations of acetic acid in cellulosic hydrolysates are often higher than those of furfural or HMF making it one of the most problematic inhibitors in the conversion of biomass to value added products [1].

*Clostridium thermocellum* is a thermophilic, anaerobic, cellulolytic Gram-positive bacterium that efficiently deconstructs and converts plant biomass to ethanol and is the most promising microorganism for the realization of consolidated bioprocessing (CBP) [2]. Pretreatment of plant biomass is still required, however, for high yields of ethanol [3]. Dilute acid pretreatment is considered as a simple and effective pretreatment method [4]. However, this pretreatment generates substantial amounts of inhibitors reaching toxic levels. Among these inhibitors are 2-furaldehyde (furfural) and 5-hydroxymethyl-2-furfural (HMF) [5]. These furan derivatives aggravate growth of microorganisms by inducing oxidative stress [6], lowering intracellular NAD(P)H levels [7], and reducing the activity of various enzymes [8] including those in the glycolytic pathway [9]. We recently showed that introduction of BdhA, a heat-stable NADPH-dependent alcohol dehydrogenase from *Thermoanaerobacter pseudethanolicus* in *C. thermocellum* increased resistance to HMF [10]. We also showed that overexpression of the endogenous spermidine synthase resulted in increased growth and increased tolerance to furans in general and that deletion of the endogenous spermidine synthase led to dramatic changes in phenotypes of *C. thermocellum* including growth and inhibitor resistance [11]. Spermidine is a low molecular weight aliphatic nitrogen compound associated with defense to diverse environmental stresses [12]. Additionally, previous studies reported that spermidine played a crucial role in the regulation of cell growth and apoptosis in addition to DNA replication, transcription, and translation [13–15].

Here we show that expression of BdhA alone had a dramatic effect on resistance to acetic acid and co-expression of the endogenous spermidine synthase resulted in increased resistance to furans and ethanol production. The most striking effect, however, was the increase in thermotolerance at 65 °C.

## Results and discussion

### Expression of BdhA alone had a dramatic effect on the tolerance of *C. thermocellum* to acetic acid

We previously explored the effect of expressing BdhA on furan tolerance in *C. thermocellum* and constructed a strain, JWCT08, that contained *bdhA* on a plasmid. However, at the time, we did not test tolerance of this *bdhA*-containing strain to acetic acid. We did not expect it to have an effect because the main activity of BdhA observed in *C. thermocellum* was NADPH-dependent reduction of HMF [10]. In this strain, the expression of BdhA was under the control of the *C. thermocellum* enolase promoter. After passage of this strain for multiple generations, we isolated a variant that had the plasmid integrated into the chromosome at the site of the enolase promoter in the *C. thermocellum* chromosome. Integration of the plasmid at that site was confirmed by PCR [10] and we compared the strains containing *bdhA* on a plasmid (JWCT06) versus integrated into the chromosome (JWCT08-1). We showed these strains to be indistinguishable with respect to acetate tolerance and fermentation products (Additional file 1: Fig. S1) and the strain containing the integrated plasmid was designated JWCT08-1 and was used in this study.

**Fig. 1:**
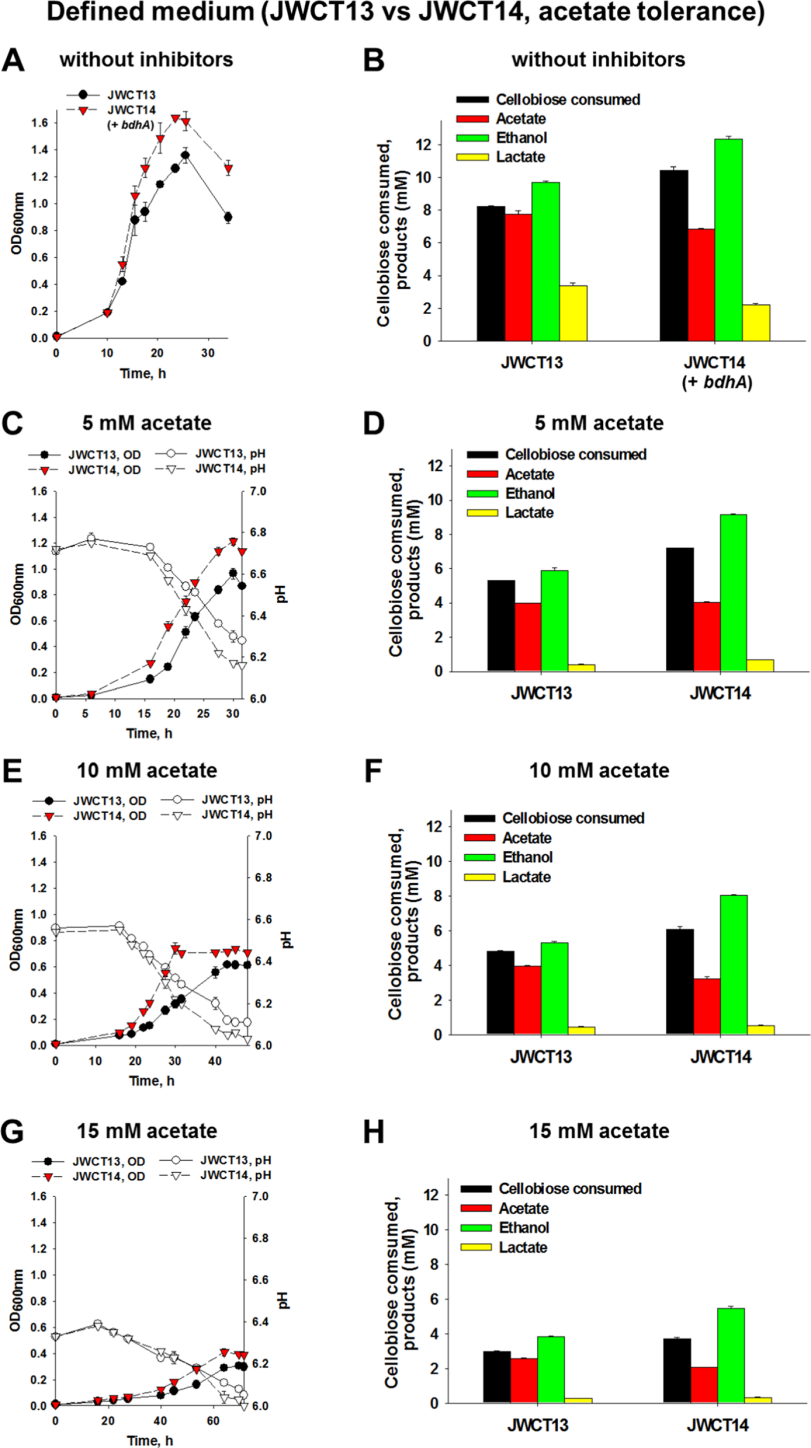
Effects of BdhA expression on cell growth and fermentation products of *C. thermocellum* strains in defined medium without fermentation inhibitors (**A** and **B**) and containing 5 (**C** and **D**), 10 (**E** and **F**), or 15 (**G** and **H**) mM acetic acid. Strains were grown in defined medium with 5 g/L cellobiose containing 10 µg/mL thiamphenicol. **A**, **C**, **E**, and **G** Cell growth of the JWCT14 strain containing *bdhA* and pJGW37 plasmid compared to the control strain. **B**, **D**, **F**, and **H** Cellobiose consumed and fermentation products of JWCT13 and JWCT16 strains. JWCT13, parental strain containing pJGW37; JWCT14, BdhA expression strain containing pJGW37. Results are the mean of duplicate experiments and error bars indicate standard deviation

Two strains were constructed for direct comparison. JWCT14 is derived from JWCT08-1 and contains *bdhA* integrated into the chromosome at the site of the enolase promoter. This strain also contains a plasmid, pJGW37, with a chloramphenicol acetyl transferase in anticipation to the need to compare with other strains used in this study. The control strain, JWCT13, does not contain *bdhA* but does contain the same plasmid, pJGW37. As shown in Fig. 1A and B, and as previously reported [10] expression of *bdhA* alone had a positive effect on growth without inhibitors and resulted in a significant increase in ethanol production. Expression of *bdhA* alone also had a dramatic effect on tolerance of *C. thermocellum* to 5 mM, 10 mM, and 15 mM acetic acid in both defined (Fig. 1C–H) and complex media (Additional file 1: Fig. S2). Specifically, in the defined medium containing 5 mM acetic acid, the maximum optical density and ethanol titer of the JWCT14 strain were 26% (*P*_value_ = 0.015) and 56% (*P*_value_ = 0.006) higher than the control strain, respectively (Fig. 1C and D). In the defined medium containing 10 mM acetic acid, maximum optical density and ethanol titer of the JWCT14 strain were 21% (*P*_value_ = 0.076) and 52% (*P*_value_ < 0.001) higher than the control strain (Fig. 1E and F). In the defined medium containing 15 mM acetic acid, the maximum optical density and ethanol titer of the JWCT14 strain were 34% (*P*_value_ = 0.037) and 43% (*P*_value_ = 0.005) higher than the control strain (Fig. 1G and H). This and earlier studies suggest that redox homeostasis in *C. thermocellum* plays an important role in its growth and tolerance to various stress conditions. Previous studies reported that acetic acid induced intracellular accumulation of reactive oxidative species (ROS) in *Saccharomyces cerevisiae* [16], and hence cell viability and ethanol production of *S. cerevisiae* in the presence of acetic acid were significantly improved by decreasing ROS levels [17]. We speculated that BdhA expression in *C. thermocellum* might result in a significant change in the overall pattern of gene expression when it is exposed to high concentrations of acetic acid. This result is consistent with previous studies which reported the upregulation of several redox-sensitive transcriptional factors whose activities rely on redox changes [18, 19].

### Spermidine supplementation to the growth medium resulted in a significant increase to resistance to acetic acid

**Fig. 2:**
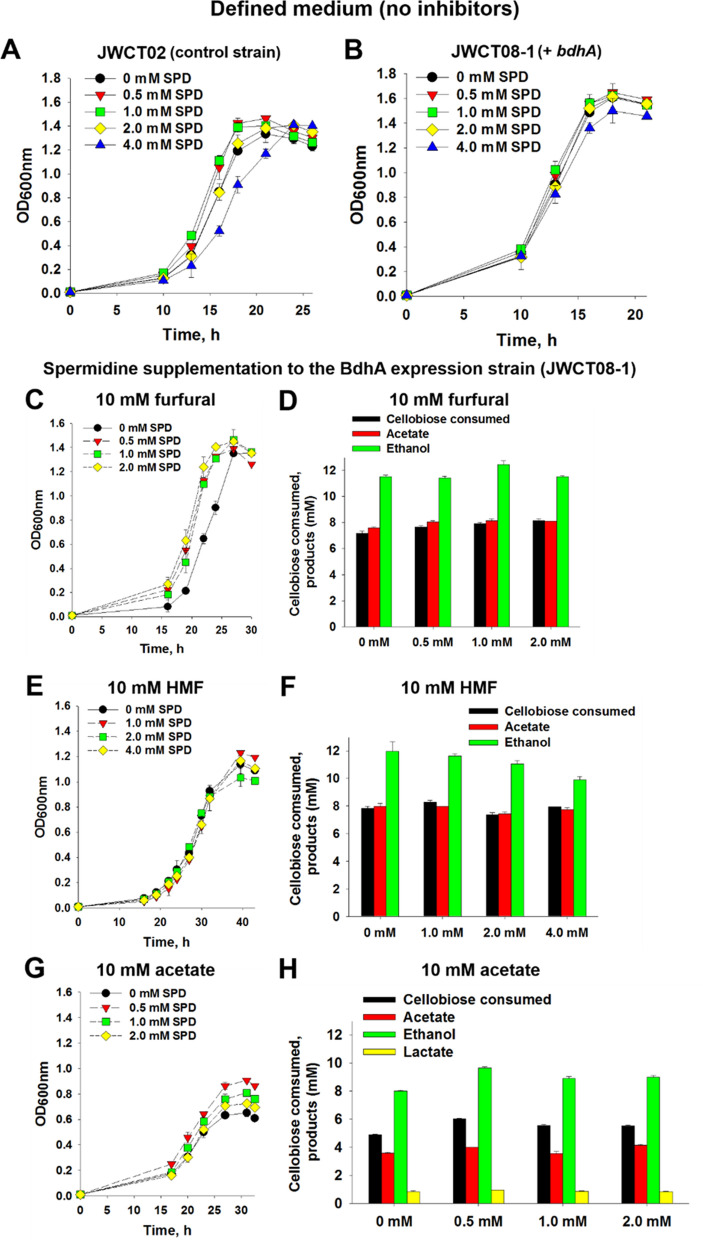
Effects of spermidine (SPD) supplementation on cell growth and fermentation products of *C. thermocellum* strains in defined medium without fermentation inhibitors (**A** and **B**) and in the presence of 10 mM furfural (**C** and **D**), HMF (**E** and **F**), or acetic acid (**G** and **H**). A Cell growth of JWCT02 (*pyrF* + pDCW89) with and without 0.5, 1.0, 2.0, and 4.0 mM spermidine. **B**, **C**, **E**, and **G** Cell growth of *C. thermocellum* JWCT08-1 (*pyrF* + *bdhA*) with and without 0.5, 1.0, 2.0, and 4.0 mM spermidine. **D**, **F**, and **H** Cellobiose consumed and fermentation products of *C. thermocellum* JWCT08-1 with and without 0.5, 1.0, 2.0, and 4.0 mM spermidine. Results are the mean of duplicate experiments and error bars indicate standard deviation

We recently showed that the addition of spermidine to the growth medium increased tolerance of *C. thermocellum* to both furfural and HMF [11]. To test whether addition of spermidine affected resistance to furans and acetic acid in the strain expressing *bdhA*, we added the same amounts previously shown to be effective [11]. Interestingly, as shown in Fig. 2C–F, exogenous addition of spermidine to the growth medium had almost no effect on resistance to furans or products generated in the presence of furan inhibitors. There was, however, a significant effect on resistance to acetic acid (Fig. 2G and H). The JWCT08-1 strain supplemented with spermidine grew to higher densities and produced significantly more ethanol than the control strain. Maximum optical densities and ethanol titers of JWCT08-1 strains supplemented with spermidine were 11–39% (*P*_value_ < 0.085) and 11–20% (*P*_value_ < 0.026) higher than the control condition. Among various spermidine concentrations added, the highest maximum optical density and ethanol titer of 0.907 ± 0.002 and 9.65 ± 0.05 mM were obtained from the strain supplemented with 0.5 mM spermidine. We note that the undissociated form of organic acids including acetic acid is much more toxic than the dissociated form and concentrations of the undissociated form increases as pH decreases. Therefore, in this study, spermidine, a basic compound, was supplemented in the form of spermidine trihydrochloride to minimize change of the medium pH. The initial pH values of all culture media was 6.40 and the pH values of media with 0, 0.5, 1, and 2 mM spermidine addition were 5.74, 5.65, 5.66, and 5.66 at the end of fermentation (Fig. 2G). Because the initial and final pH values of culture media with and without spermidine addition were almost identical, we conclude that the addition of spermidine resulted in a significant increase in resistance to acetic acid of which an underlying mechanism is independent of pH.

### Co-expression of SpeE and BdhA was synergistic on growth and resistance to furans and acetic acid

We recently reported that introduction of *bdhA* into *C. thermocellum* resulted in increased tolerance to HMF [10] and that overexpression of the endogenous spermidine synthase (SpeE) in *C. thermocellum* resulted in increased tolerance to both furans and increased ethanol production [11]. To test whether co-expression of these proteins in *C. thermocellum* would have a synergistic effect we constructed a strain, JWCT16, containing *bdhA* and simultaneously overexpressing the endogenous spermidine synthase. The plasmid containing the spermidine expression cassette and a chloramphenicol acetyltransferase (*cat*) gene (pSKW59) was transformed into strain JWCT08-1 containing an integrated copy of the *bdhA* gene. Transformants were selected for thiamphenicol resistance [11]. The presence of this plasmid in transformants was confirmed by PCR analysis, and structural stability during transformation and replication in *C. thermocellum* was confirmed by restriction analysis of pSKW59 plasmid DNA before and after transformation of *C. thermocellum* and back-transformation to *E. coli* (Additional file 1: Fig. S3).

**Fig. 3:**
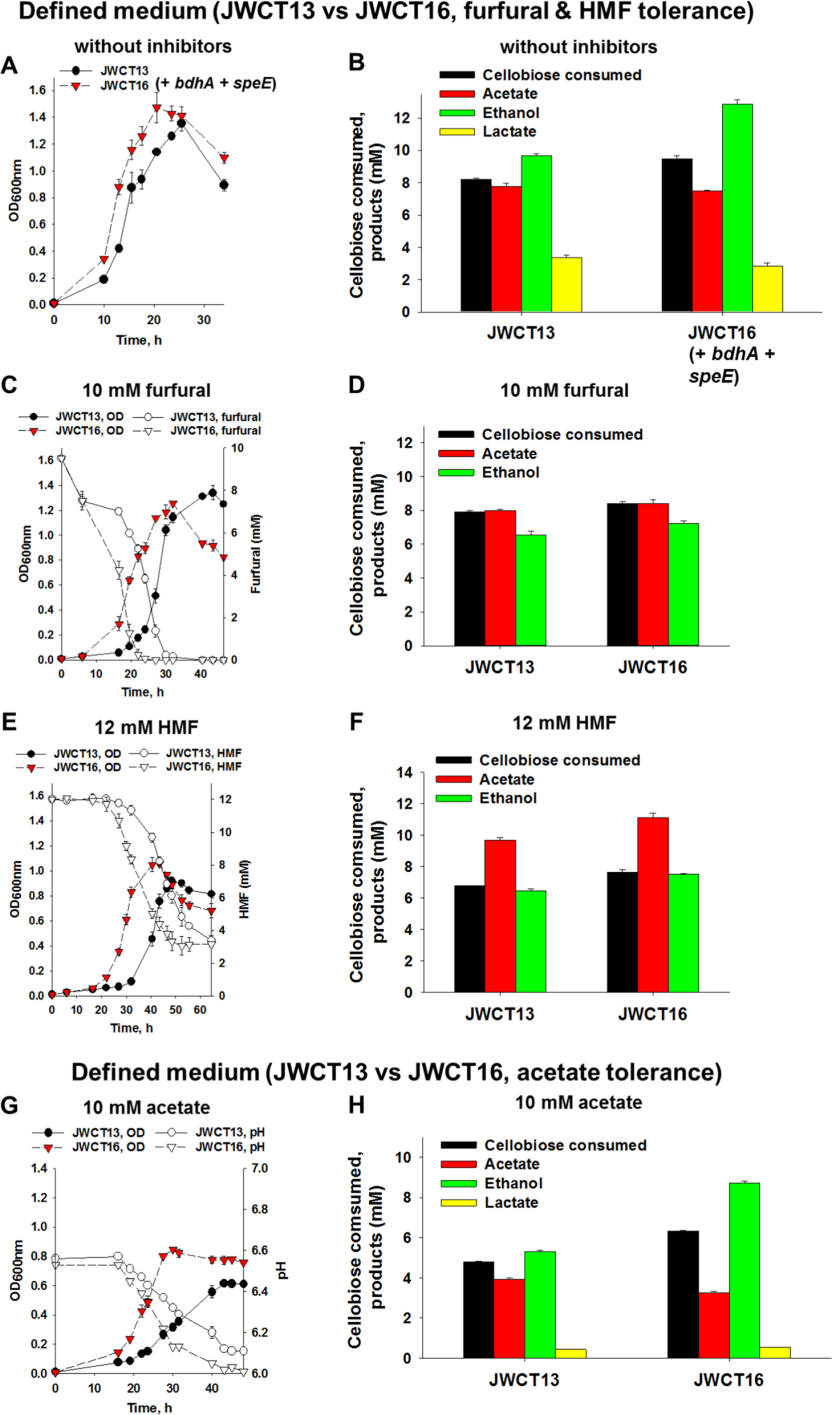
Effects of SpeE expression with the BdhA expression on cell growth and fermentation products of *C. thermocellum* strains in defined medium without fermentation inhibitors (**A** and **B**) and in the presence of 10 mM furfural (**C** and **D**), 12 mM HMF (**E** and **F**), or 10 mM acetic acid (**G** and **H**). Strains were grown in defined or complex medium with 5 g/L cellobiose containing 10 µg/mL thiamphenicol. **A**, **C**, **E**, and **G** Cell growth of JWCT13 (*pyrF* + pJGW37) and JWCT16 (*pyrF* + *bdhA* + *speE*) strains. **B**, **D**, **F**, and **H** Cellobiose consumed and fermentation products of JWCT13 and JWCT16 strains. JWCT13, parental strain; JWCT16, BdhA and SpeE expressing strain. Results are the mean of duplicate experiments and error bars indicate standard deviation

As sown in Fig. 3A and B, co-expression of SpeE and BdhA had a positive effect on growth without inhibitors and resulted in an increase in ethanol production. Batch fermentations of *C. thermocellum* JWCT13 (the parental strain) and JWCT16 (BdhA and SpeE expressing strain) were performed in both the defined and complex media containing 10 mM furfural or 12 mM HMF (Additional file 1: Fig. S4). As shown in Fig. 3C–H, there was a positive effect of SpeE and BdhA on resistance to furans or products generated in the presence of furan inhibitors. In defined medium, conversion of HMF was rapid in the JWCT16 strain, resulting in 15% (*P*_value_ < 0.001) higher maximum optical density and 16% (*P*_value_ = 0.007) higher ethanol titer than those of the control strain (Fig. 3E and F). Similar results were observed in the complex medium.

Co-expression of SpeE and BdhA had a dramatic positive effect on growth in the presence of acetate. As shown in Fig. 3G and H and Additional file 1: Fig. S5, engineered strains exhibited shorter lag-phase periods and higher maximum optical densities than those of the control strain in the presence of 5, 10 or 15 mM acetic acid as well as significantly increased levels of ethanol production. In the defined medium containing 5 mM acetic acid, maximum optical density and ethanol titer of the JWCT16 strain were 27% (*P*_value_ = 0.014) and 68% (*P*_value_ = 0.001) higher than the control strain (Additional file 1: Fig. S5A and D). In the defined medium containing 10 mM acetic acid, maximum optical density and ethanol titer of the JWCT16 strain were 38% (*P*_*value*_ = 0.003) and 64% (*P*_*value*_ = 0.001) higher than the control strain (Fig. 3G and H). In the defined medium containing 15 mM acetic acid, maximum optical density and ethanol titer of the JWCT16 strain were 24% (*P*_value_ = 0.018) and 40% (*P*_value_ = 0.001) higher than the control strain (Additional file 1: Fig. S5C and F). Similar results were observed in the complex medium.

### The combined expression of spermidine synthase and BdhA resulted in increased tolerance to higher temperature

**Fig. 4:**
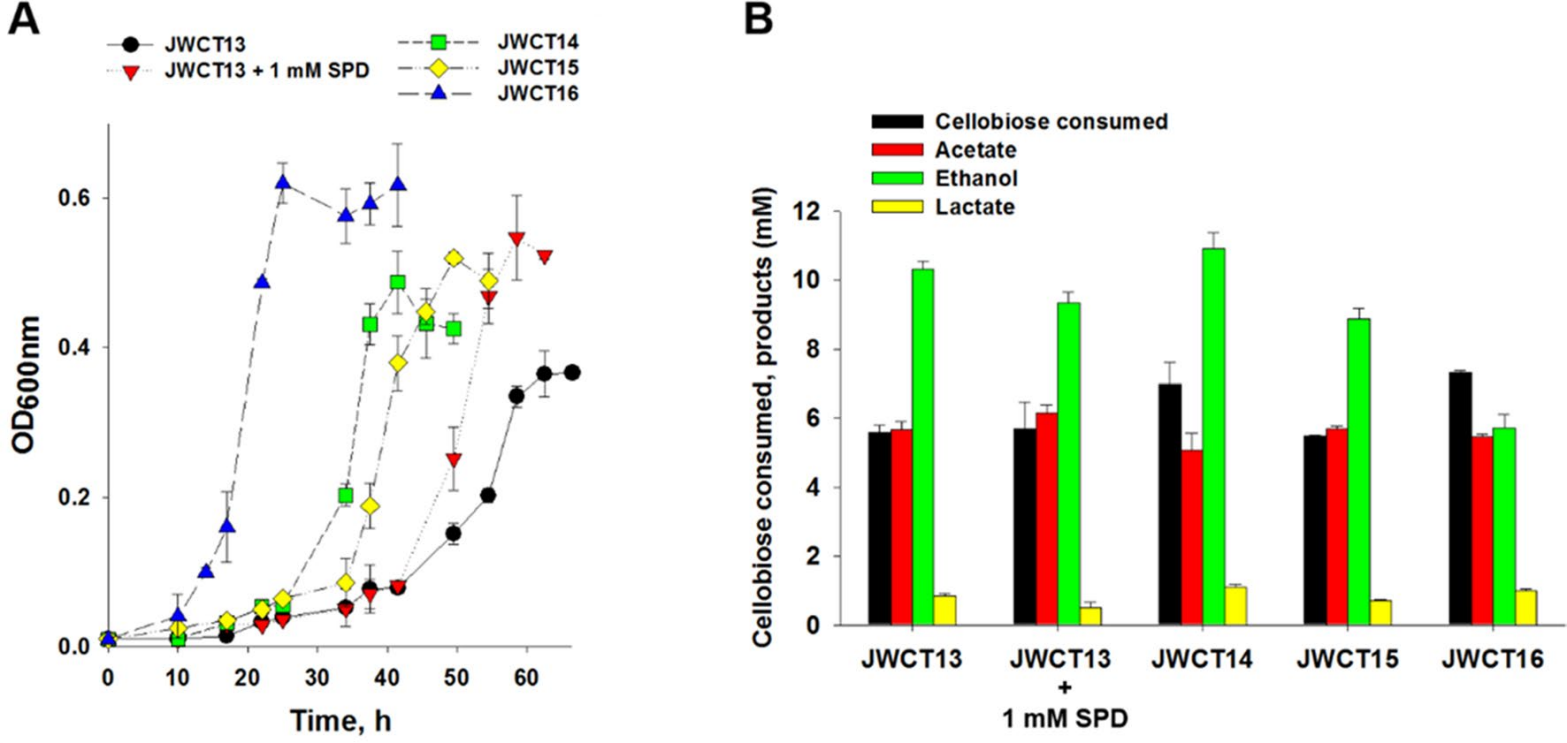
Effects of SpeE expression with the BdhA expression on growth of *C. thermocellum* at 65 °C. **A** and **B** Cell growth (**A**) and cellobiose consumed and fermentation products (**B**) of JWCT13 with and without 1 mM spermidine, JWCT14, JWCT15, and JWCT16 strains in defined medium with 5 g/L cellobiose containing 10 µg/mL thiamphenicol. JWCT13, parental strain; JWCT14, BdhA expressing strain; JWCT15, SpeE expressing strain; JWCT16, BdhA and SpeE expressing strain. Results are the mean of duplicate experiments and error bars indicate s.d

To test the effect of co-expression of SpeE and BdhA on temperature tolerance, we first compared growth of *C. thermocellum* JWCT13 (the parental strain), with and without 1 mM spermidine at 65 °C in defined medium containing 10 µg/mL thiamphenicol. As shown in Fig. 4A, JWCT13 exhibited a prolonged lag-phase period and low maximum cell density at 65 °C, addition of 1 mM spermidine resulted in a 49% (*P*_value_ = 0.069) higher maximum cell density. In the same experiment, we tested JWCT14 (the BdhA expression strain), JWCT15 (the SpeE expression strain), and JWCT16 (the BdhA and SpeE expressing strain) for comparison. All three engineered strains, grew faster and reached higher cell densities than the control strain at 65 °C (Fig. 4A) producing similar amounts of ethanol except for JWCT15 and JWCT16 that produced significantly less ethanol (Fig. 4B). Among them, co-expression of BdhA and SpeE (JWCT16) resulted in the highest maximum optical density of 0.62 ± 0.019, which was 69% (*P*_value_ = 0.023) higher than that of the control strain (Fig. 4A). The robust strains constructed in this study can be applied for producing ethanol at temperatures near the boiling point of ethanol, in which ethanol produced can be collected economically from the aqueous phase.

## Conclusions

We previously reported that expression of BdhA in *C. thermocellum* resulted not only in increased resistance to HMF, but also improved growth on cellulosic substrates and ethanol production [10]. In this study, we observed that BdhA expression also resulted in improved tolerance of *C. thermocellum* to acetic acid.

These engineered strains exhibiting distinct improved phenotypes against fermentation inhibitors can serve as customized host strains for fermenting cellulosic hydrolysates containing different compositions of fermentation inhibitors. In the case of utilizing lignocellulose hydrolysates containing only furan derivatives or acetic acid, the JWCT16 strain exhibiting the highest tolerance against furfural and acetic acid could be used. On the other hand, in the case of utilizing lignocellulose hydrolysates containing furfural, HMF, and acetic acid, the JWCT15 strain showing improved tolerance to furan derivatives and acetic acid simultaneously [11] is applicable. The strains developed in this study can have broad applications not only to produce ethanol, but also many other biochemicals and biofuels from lignocellulosic biomass.

## Materials and methods

### Bacterial strains, media, and culture conditions

*C. thermocellum* and *E. coli* strains used in this study are listed in Table 1. All *C. thermocellum* strains were grown anaerobically in modified CTFUD medium [20], pH 7.0, with cellobiose (0.5% w/v) as the sole carbon source for routine growth and transformation experiments. Defined medium, CTFUD-NY [20], contained a vitamin mix of *p*-aminobenzoic acid, vitamin B12, biotin, and pyridoxamine in place of the yeast extract. For uracil auxotrophs, the medium was supplemented with 40 µM uracil. Complex medium contained the vitamin mix, casein (0.2% w/v) and yeast extract (0.045% w/v), referred to as CTFUD + C. *C. thermocellum* cells were grown at 60 °C, under an atmosphere of 85% nitrogen, 10% CO_2_, and 5% hydrogen. *E. coli* BL21 (Invitrogen, Grand Island, NY, USA) was grown in LB medium with 50 μg/mL apramycin for plasmid selection. Plasmid DNA was isolated using a Qiagen Miniprep Kit (Qiagen, Valencia, CA, USA). Chromosomal DNA from *C. thermocellum* strains was extracted using the Quick-gDNA MiniPrep (Zymo Research, Irvine, CA, USA) according to the manufacturer’s instructions. Restriction enzymes (New England BioLabs, Ipswich, MA, USA), and the Fast-link DNA ligase kit (Epicentre Biotechnologies, Madison, WI, USA) were used according to the manufacturer’s instructions. Construction of the spermidine expression cassette was previously reported [11]. PCR amplification with primers DC460 (specific for plasmid pSKW59) and SK166 (specific for SpeE) were used to confirm co-expression of SpeE with BdhA. Primers used for plasmid constructions and confirmation are listed in Table S1. Electrotransformation of *C. thermocellum* cells was performed as previously described [21]. Cultures, electro-pulsed with plasmid DNA (~ 0.4 μg), were recovered in CTFUD + C medium at 60 °C, plated on solid CTFUD + C medium containing 10 µg/mL thiamphenicol (Sigma, St. Louis, MO) to obtain isolated colonies, and DNA was isolated from transformants. Taq polymerase (Sigma, St. Louis, MO, USA) was used for PCR reactions to confirm the presence of the plasmid. PCR amplification with primers DC460 (specific for plasmid pSKW59) and SK166 (specific for SpeE) was used to confirm the presence of the plasmid with the SpeE.

**Table 1:** Strains and plasmids used in this study

Name	Description	References
Strains		
LL1005	*C. thermocellum* DSM 1313 *pyrF* (*ura*^*−*^/5-FOA^R^)	[21]
JWCT02	LL1005 containing pDCW89 (*ura*^+^/5-FOA^S^)	[21]
JWCT06	LL1005 containing pSKW01 (*ura*^+^/5-FOA^S^)	[10]
JWCT08-1	LL1005*::*P_enolase_—*bdhA* containing pJGW37 (*ura*^+^/5-FOA^S^)	[10]
JWCT13	LL1005 containing pJGW37 (*ura*^*−*^/5-FOA^R^/Tm^R^)	[11]
JWCT14	JWCT08-1 containing pJGW37 (*ura*^+^/5-FOA^S^/Tm^R^)	This study
JWCT15	LL1005 containing pSKW59 (*ura*^*−*^/5-FOA^R^ /Tm^R^)	[11]
JWCT16	JWCT08-1 containing pSKW59 (*ura*^+^/5-FOA^S^/Tm^R^)	This study
Plasmids		
pDCW89	*E. coli*/*C. thermocellum* shuttle vector P_Cbes2105_ -*pyrF*	[21, 22]
pJGW37	*E. coli*/*C. thermocellum* shuttle vector P_Cbes2105_ -*cat*	[21]
pSKW01	Expression vector P_Cbes2105_—*pyrF* and P_S-layer_ -*bdhA*	[10]
pSKW59	Expression vector P_Cbes2105_—*cat* and P_S-layer_ -*speE*	[11]

### Fermentations

Cultures JWCT02 or JWCT08-1 were serially passaged every 24 h in 20 mL CTFUD-NY medium without uracil. After the second transfer, cultures were inoculated to the last culture to initial optical density (OD_600_) of 0.01. Batch fermentations were performed at 60 °C without agitation in 20 mL CTFUD-NY medium without uracil containing 10 mM furfural, HMF, or acetic acid. Cultures of JWCT13, JWCT14, JWCT15 or JWCT16 were first sub-cultured in 20 mL CTFUD-NY medium with uracil containing 10 µg/mL thiamphenicol, and then transferred to 20 mL CTFUD + C medium containing 10 µg/mL thiamphenicol. Pre-cultured cells from CTFUD + C medium were inoculated into cultures with initial optical density (OD_600_) of 0.01 for fermentations of media containing one of the three inhibitors (furfural, HMF, and acetic acid). Optical cell density was monitored using a Jenway Genova spectrophotometer, measuring absorbance at 600 nm.

### Analytical methods

Cellobiose, glucose, acetate, lactate, ethanol, HMF, and furfural concentrations were determined by high-performance liquid chromatography (HPLC, Agilent Technologies 1200 Series). Metabolites were separated on an Aminex HPX-87H column (Bio-Rad Laboratories) at isocratic temperature (50 °C) and a flow (0.6 mL/min) rate in 5.0 mM H_2_SO_4_, and then passed through a refractive index detector (Agilent 1200 Infinity Refractive Index Detector). Peak areas and retention times were compared to known standards of the same analyte.

## Supplementary Information

**Additional file: 1: Table S1.** List of primers used in this study. **Figure S1.** Effects of BdhA expression on tolerance of *C. thermocellum* to acetic acid. **Figure S2.** Effects of BdhA expression on cell growth and fermentation products of *C. thermocellum* strains in complex medium without fermentation inhibitors (A and B) and containing 5 (C and D), 10 (E and F), or 15 (G and H) mM acetic acid. **Figure S3**. Verification of stable presence of pSKW59 plasmid in *C. thermocellum* transformants. **Figure S4**. Effects of SpeE expression with the BdhA expression on cell growth in defined (A-F) or complex medium (G-L) without fermentation inhibitors (A, D, G, and J) and tolerance to furfural (B, E, H, and K) and HMF (C, F, I, and L). **Figure S5.** Effects of SpeE expression with the BdhA expression on cell growth and fermentation products of *C. thermocellum* strains in defined (A-F) or complex medium (G-L) containing 5, 10, or 15 mM acetic acid.

## Data Availability

Plasmids and strains generated in this study are available upon request.

## Abbreviations

CBP: Consolidated bioprocessing
Furfural: 2-Furaldehyde
HMF: 5-Hydroxymethyl-2-furfural
BdhA: A heat-stable NADPH-dependent alcohol dehydrogenase from *Thermoanaerobacter pseudethanolicus*
SpeE: Spermidine synthase
CAT: Chloramphenicol acetyltransferase
LB broth: Luria–Bertani broth
OD: Optical density
HPLC: High-performance liquid chromatography
