# Supplementary material for: Simultaneous expression of an endogenous spermidine synthase and a butanol dehydrogenase from Thermoanaerobacter pseudethanolicus in Clostridium thermocellum results in increased resistance to acetic acid and furans, increased ethanol production and an increase in thermotolerance
Source: Biotechnol Biofuels Bioprod. 2023 Mar 14;16:46. doi: 10.1186/s13068-023-02291-6 (PMC10012442; doi:10.1186/s13068-023-02291-6)
Supplement: Supplementary file 1 — Additional file: 1: Table S1. List of primers used in this study. Figure S1. Effects of BdhA expression on tolerance of C. thermocellum to acetic acid. Figure S2. Effects of BdhA expression on cell growth and fermentation products of C. thermocellum strains in complex medium without fermentation inhibitors (A and B) and containing 5 (C and D), 10 (E and F), or 15 (G and H) mM acetic acid. Figure S3. Verification of stable presence of pSKW59 plasmid in C. thermocellum transformants. Figure S4. Effects of SpeE expression with the BdhA expression on cell growth in defined (A-F) or complex medium (G-L) without fermentation inhibitors (A, D, G, and J) and tolerance to furfural (B, E, H, and K) and HMF (C, F, I, and L). Figure S5. Effects of SpeE expression with the BdhA expression on cell growth and fermentation products of C. thermocellum strains in defined (A-F) or complex medium (G-L) containing 5, 10, or 15 mM acetic acid. [file 13068_2023_2291_MOESM1_ESM.docx]

**Supplementary data**

Table S1. List of primers used in this study. The italicized sequences indicate the recognition sites of the corresponding restriction enzymes.

| Name | Sequence (5’ → 3’) | Restriction enzyme | Description |
| --- | --- | --- | --- |
| DC460 | AGAGAG*CGATCG*ACAGTTTGATTACAGTTTAGTCAGAGCT | PvuI | Confirm transformants |
| SK166 | AGA*GCATGC*TTATTTTATTAATTCTTTTGCAAACTTCGGCAGT | SphI |  |


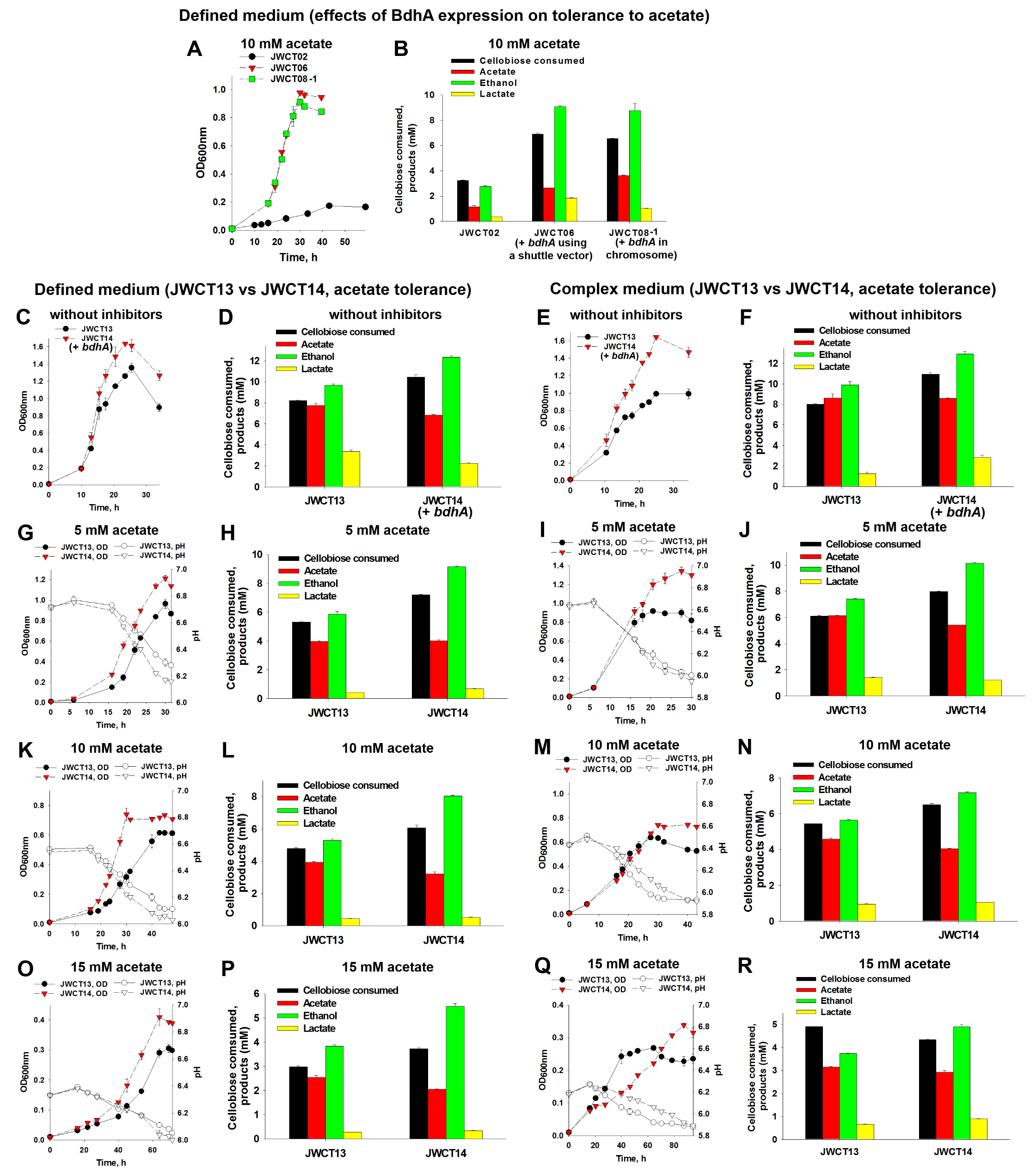


**Figure S1.** **Effects of BdhA expression on tolerance of *C. thermocellum* to acetic acid.** Strains were grown in defined medium containing 10 mM acetic acid. Cell growth (A) and cellobiose consumed and fermentation products (B) of strains expressing BdhA compared to the control strain. JWCT02 (*△pyrF* + pDCW89), the parent control strain; JWCT06 (*△pyrF* + *bdhA*), containing P_S-layer_ - *bdhA*; JWCT08-1 (*△pyrF* + *bdhA*), containing P_enolase_ - *bdhA*.


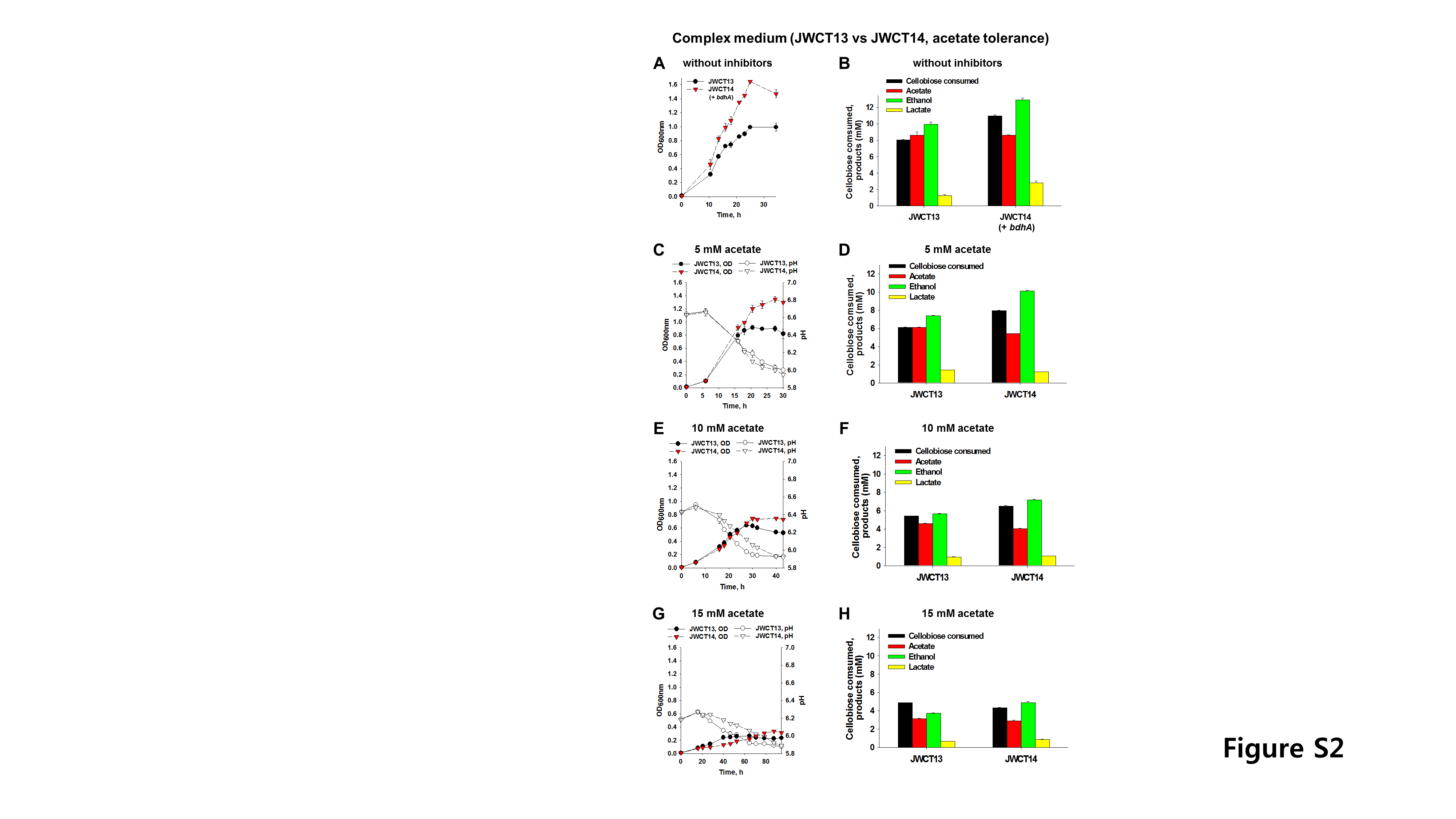


**Figure S2.** **Effects of BdhA expression on cell growth and fermentation products of *C. thermocellum* strains in complex medium without fermentation inhibitors (A and B) and containing 5 (C and D), 10 (E and F), or 15 (G and H) mM acetic acid.** Strains were grown in complex medium with 5 g/L cellobiose containing 10 µg/mL thiamphenicol. (A, C, E, and G) Cell growth of the JWCT14 strain containing *bdhA* and pJGW37 plasmid compared to the control strain. (B, D, F, and H) Cellobiose consumed and fermentation products of JWCT13 and JWCT16 strains. JWCT13, parental strain containing pJGW37; JWCT14, BdhA expression strain containing pJGW37. Results are the mean of duplicate experiments and error bars indicate standard deviation.


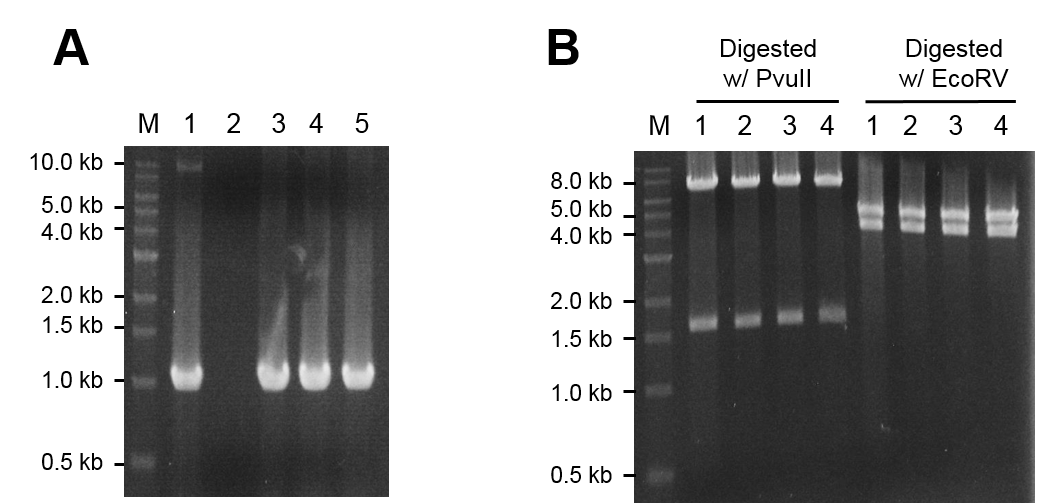


**Figure S3**. **Verification of stable presence of pSKW59 plasmid in *C. thermocellum* transformants.** (A) Gel showing the 1.0 kb PCR products using plasmid-specific primers DC460 and SK166. 1: plasmid pSKW59; 2: negative control LL1005; 3, 4 and 5: JWCT16 (JWCT08-1 *+* pSKW59). (B) Restriction analysis of pSKW59 plasmid DNA before and after transformation of *C. thermocellum* JWCT16 and back-transformation to *E. coli*. 1: plasmid DNA isolated from *E. coli* BL21, digested with either PvuII (7.0 kb and 1.6 kb cleavage products), or with EcoRV (4.7 and 3.9 kb cleavage products); 2, 3, and 4; plasmid DNA isolated from three biologically independent *E. coli* BL21 back-transformants using total DNA isolated from *C. thermocellum* transformants, digested with either PvuII or EcoRV; M: NEB 1 kb DNA ladder.


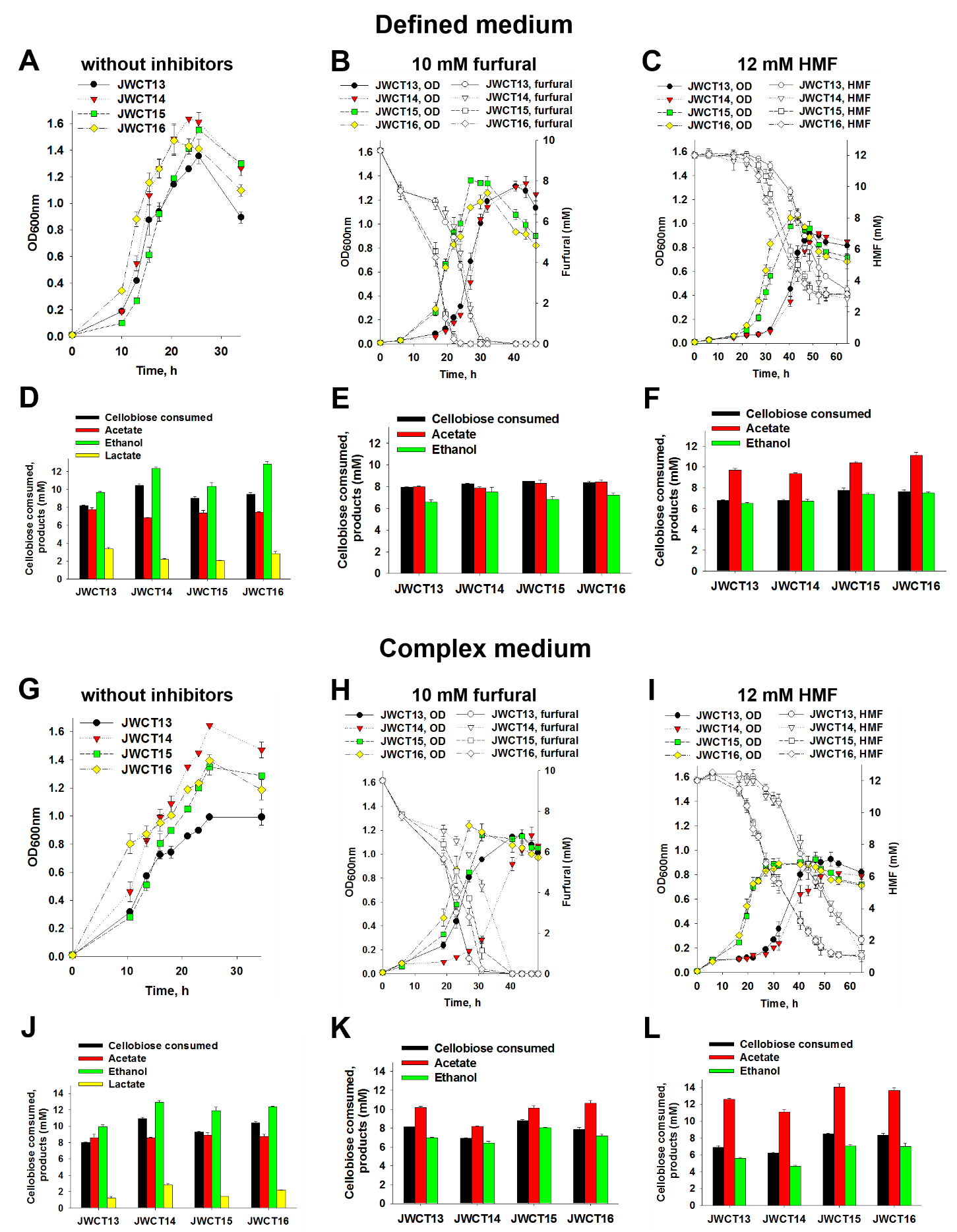


**Figure S4**. **Effects of SpeE expression with the BdhA expression on cell growth in defined (A-F) or complex medium (G-L) without fermentation inhibitors (A, D, G, and J) and tolerance to furfural (B, E, H, and K) and HMF (C, F, I, and L).** Strains were grown in defined or complex medium with 5 g/L cellobiose containing 10 µg/mL thiamphenicol. (A, B, C, G, H, and I) Cell growth of JWCT13, JWCT14, JWCT15, and JWCT16 strains (D, E, F, J, K, and L) Cellobiose consumed and fermentation products of JWCT13, parental strain; JWCT14, BdhA expressing strain; JWCT15, SpeE expressing strain; JWCT16, BdhA and SpeE expressing strain. Results are the mean of duplicate experiments and error bars indicate s.d.

**
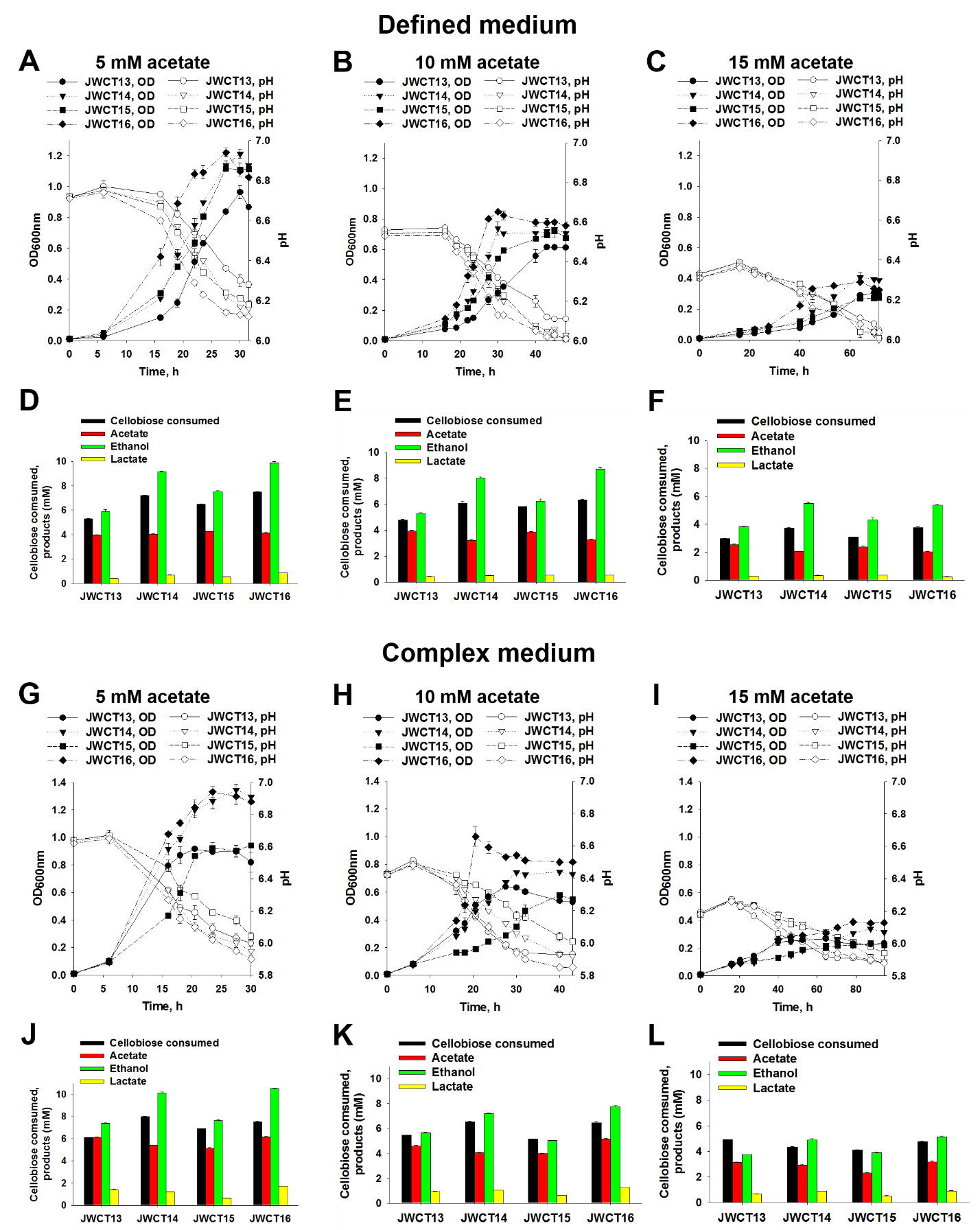
**

**Figure S5. Effects of SpeE expression with the BdhA expression on cell growth and fermentation products of *C. thermocellum* strains in defined (A-F) or complex medium (G-L) containing 5, 10, or 15 mM acetic acid.** (A, B, C, G, H, and I) Batch fermentations of JWCT13, JWCT14, JWCT15, and JWCT16 strains in medium with 5 g/L cellobiose and 10 µg/mL thiamphenicol containing 5 (A and G), 10 (B and H), or 15 (C and I) mM acetic acid. (D, E, F, J, K, and L) Cellobiose consumed and fermentation products of JWCT13, JWCT14, JWCT15, and JWCT16 strains in the medium containing 5 (D and J), 10 (E and K), or 15 (F and L) mM acetic acid. JWCT13, parental strain; JWCT14, BdhA expressing strain; JWCT15, SpeE expressing strain; JWCT16, BdhA and SpeE expressing strain. Results are the mean of duplicate experiments and error bars indicate s.d.
